# A review on synthesis and antibacterial potential of bio-selenium nanoparticles in the food industry

**DOI:** 10.3389/fmicb.2023.1229838

**Published:** 2023-07-13

**Authors:** Bo Ao, Qingquan Du, Decheng Liu, Xiaoshan Shi, Junming Tu, Xian Xia

**Affiliations:** Hubei Key Laboratory of Edible Wild Plants Conservation & Utilization, Hubei Engineering Research Center of Characteristic Wild Vegetable Breeding and Comprehensive Utilization Technology, Huangshi Key Laboratory of Lake Environmental Protection and Sustainable Utilization of Resources, Hubei Normal University, Huangshi, China

**Keywords:** Bio-SeNPs, synthesis, antibacterial, foodborne pathogens, toxicity

## Abstract

Effective control of foodborne pathogen contamination is a significant challenge to the food industry, but the development of new antibacterial nanotechnologies offers new opportunities. Notably, selenium nanoparticles have been extensively studied and successfully applied in various food fields. Selenium nanoparticles act as food antibacterial agents with a number of benefits, including selenium as an essential trace element in food, prevention of drug resistance induction in foodborne pathogens, and improvement of shelf life and food storage conditions. Compared to physical and chemical methods, biogenic selenium nanoparticles (Bio-SeNPs) are safer and more multifunctional due to the bioactive molecules in Bio-SeNPs. This review includes a summarization of (1) biosynthesized of Bio-SeNPs from different sources (plant extracts, fungi and bacteria) and their antibacterial activity against various foodborne bacteria; (2) the antibacterial mechanisms of Bio-SeNPs, including penetration of cell wall, damage to cell membrane and contents leakage, inhibition of biofilm formation, and induction of oxidative stress; (3) the potential antibacterial applications of Bio-SeNPs as food packaging materials, food additives and fertilizers/feeds for crops and animals in the food industry; and (4) the cytotoxicity and animal toxicity of Bio-SeNPs. The related knowledge contributes to enhancing our understanding of Bio-SeNP applications and makes a valuable contribution to ensuring food safety.

## 1. Introduction

Foodborne pathogens are a main cause of foodborne illness and food poisoning, which are food safety issues with serious implications for human health and economic development. According to the World Health Report, millions of hospital cases occur due to food contamination, and hundreds of thousands die due to foodborne diseases every year (Wei and Zhao, 2021). Globally, foodborne illnesses are normally caused by 31 major pathogens (Riley, 2020), most of which are bacterial pathogens, which can result in intoxication, infection and toxicoinfections (Abebe et al., 2020). Usually, the clinical syndromes of foodborne bacterial infection are fever, mild diarrhea, headaches, vomiting, muscle cramps, abdominal pain and even more complex illnesses (Iwu and Okoh, 2019). The potential risk of foodborne bacteria is commonly present in various foods during production, packaging, and transportation (Xing et al., 2022). The ingestion of foodborne pathogenic bacteria contaminated foods, such as seafoods (Ali et al., 2020), milk and dairy products (Keba et al., 2020), meat and meat products (Zhao et al., 2022), raw and ready-to-eat green leafy vegetables (Azimirad et al., 2021) and grains (such as rice, noodles, and rice noodles) (Li et al., 2020), might lead to serious foodborne diseases. The increasing incidences of foodborne diseases (Hoffmann and Scallan Walter, 2020) and product corruption (Gurtler and Gibson, 2022) cause serious economic losses and significantly hinder social development, which has brought food safety to the forefront of public health concerns.

Currently, as standards of life have improved, one of the most serious challenges for the food industry is to ensure food safety while also ensuring food quality (Yan et al., 2021). To ensure food safety, thermal sterilization is the most common method for inactivating foodborne pathogens, but high temperatures also diminish the quality of food products (Guo et al., 2022). Although nonthermal physical technologies have emerged in recent years to improve the quality of food products, their application has been severely limited by their high cost and technical threshold (Chacha et al., 2021; Khouryieh, 2021). Chemical bacteriostatic agents, including antibiotics, are the most common method for inhibiting the growth of bacteria in livestock, aquaculture and agriculture (Chen et al., 2020; Shao et al., 2021; Tadic et al., 2021). However, this method leads to the emergence of residual antibiotics and multiple foodborne drug-resistant bacteria in the food chain, posing a serious threat to food safety (Liao et al., 2020; Thapa et al., 2020). Various foodborne bacteria, such as *Escherichia coli* (MDR) (Liu et al., 2022), *Staphylococcus aureus* (MRSA) (Algammal et al., 2020), *Salmonella enterica* serovar Rissen (Xu et al., 2020) and *Listeria monocytogenes* (Baquero et al., 2020), have been reported to exhibit drug resistance. These bacteria have evolved multiple mechanisms, including reduced cell membrane permeability, efflux pump mechanisms, target site mutation mechanisms, and enzymatic hydrolysis, to cope with antibiotics (Ge et al., 2022). Consequently, innovative technological approaches are urgently required to combat foodborne pathogens.

In recent years, nano antimicrobial agents have attracted researchers’ attention (Fatima et al., 2021). In the food industry, selenium nanoparticles, as an alternative antimicrobial agent, have many benefits over other nanomaterials, such as (1) selenium as a trace element in food (Kieliszek, 2019), (2) prevention of drug resistance induction in foodborne pathogens (Truong et al., 2021), and (3) improvement of shelf life and food storage conditions (Ndwandwe et al., 2020; Salem M. F. et al., 2022). Selenium nanoparticles are normally synthesized by physical, chemical and biological methods (Nayak et al., 2021) in which Se(IV) can be reduced to Se(0) and then form SeNPs (Zambonino et al., 2021). However, compared to other conventional physical and chemical methods, microbial and plant-mediated synthesis of biogenic selenium nanoparticles (Bio-SeNPs) with various bioactive substances have extensive biological applications (Vijayakumar et al., 2022). In addition, Bio-SeNPs also have the advantages of high biocompatibility, eco-friendliness and low toxicity (Ikram et al., 2021). At the same time, numerous studies have also demonstrated the excellent antibacterial activity of Bio-SeNPs against food-borne pathogens (Abu-Elghait et al., 2021; Zhang et al., 2021; Salem S. S. et al., 2022). Additionally, numerous studies have demonstrated the low/nontoxicity of Bio-SeNPs at the cellular and animal levels (Majeed et al., 2020; Perumal et al., 2021; Souza et al., 2022). Overall, Bio-SeNPs have value in improving food safety against food pathogens in the food industry.

In this review, the target references were searched using Google Scholar database and the selected keywords were “biosynthesis + selenium nanoparticles + antibacterial/food/toxicity.” We focus on the new research breakthroughs of Bio-SeNPs, including (1) the biosynthesis methods and antibacterial activity of Bio-SeNPs; (2) the mechanisms of Bio-SeNPs against foodborne pathogens; (3) the potential application of Bio-SeNPs in the food industry; and (4) the toxicity of Bio-SeNPs.

## 2. Bio-SeNPs antibacterial activity against foodborne pathogens

**Figure 1:**
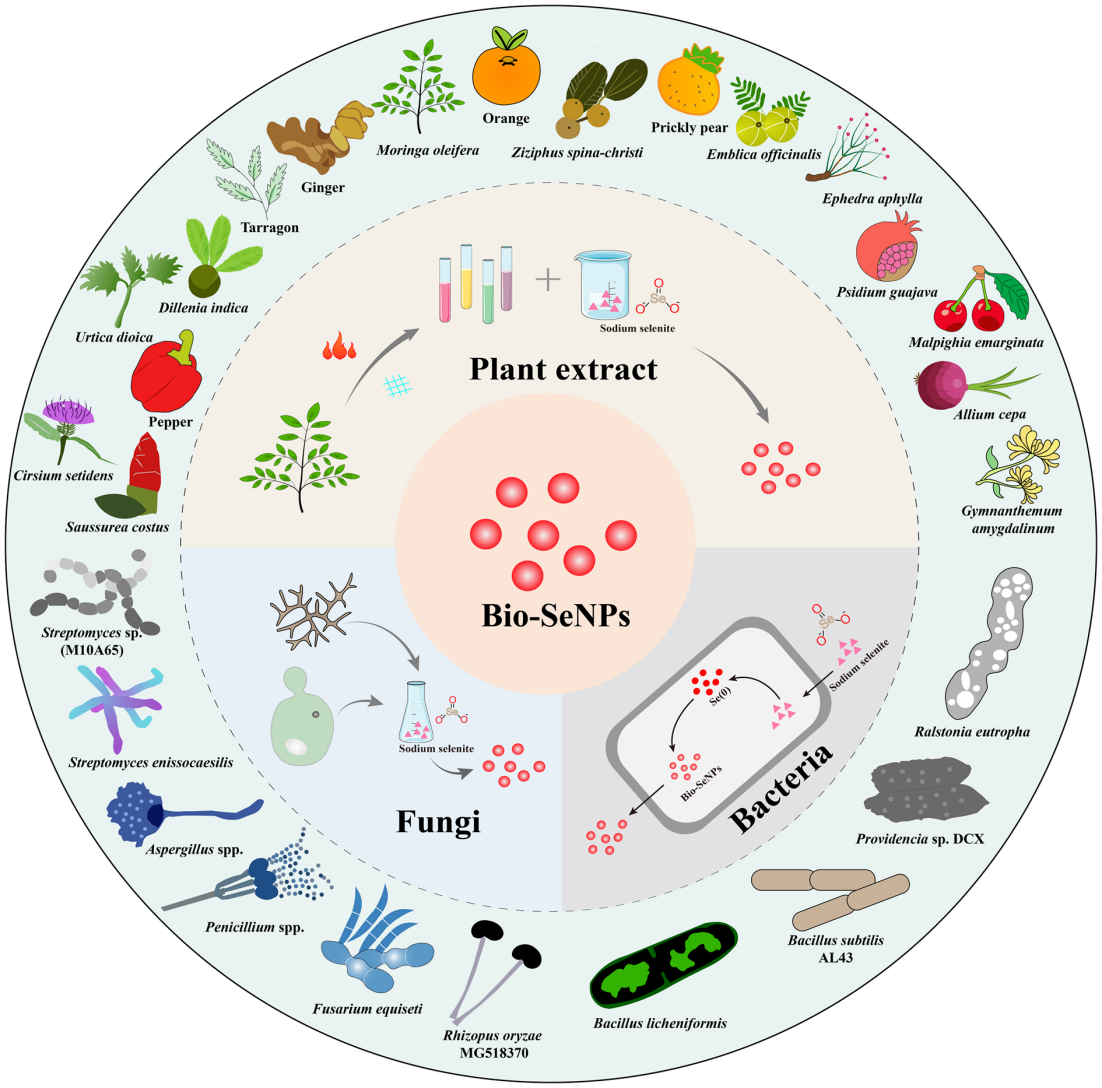
The technical routes for Bio-SeNPs synthesis based on plants extracts, bacteria, and fungi (inner ring) and some representative plants, bacteria and fungi used in the synthesis of Bio-SeNPs (outer ring).

Biological approaches to the synthesis of selenium nanoparticles arose from the need to develop new and environmentally friendly antibacterial agents. Numerous studies have shown that Bio-SeNPs have excellent antibacterial capacity and inhibit foodborne pathogens by various antibacterial mechanisms. Usually, Bio-SeNPs are synthesized from bacterial, fungal, and plant extracts (Shoeibi et al., 2017). Figure 1 illustrates the various sources of synthetic Bio-SeNPs against foodborne pathogens. These bioderived selenium nanoparticles have more potential applications in the food industry due to their excellent antibacterial activity and safety (Ndwandwe et al., 2020). Table 1 summarizes the reported Bio-SeNPs and their antibacterial properties against foodborne pathogens, such as *S. aureus*, *E. coli*, *L. monocytogenes*, *Salmonella*, *Bacillus cereus*, and *Alicyclobacillus acidoterrestris*.

**Table 1:** The antibacterial activities against foodborne pathogens of Bio-SeNPs.

Biological model		Characteristics	Foodborne pathogens	Concentrations (μg/mL)	References
Plant extracts	*Allium cepa + Malpighia emarginata*	Spherical, 245–321 nm	*Escherichia coli* ATCC 25922	196	Souza et al. (2022)
			*Staphylococcus aureus* ATCC 25923	24.5	
			BEC 9393 (MRSA)	49	
	*Gymnanthemum amygdalinum + Malpighia emarginata*	Spherical, 245–321 nm	*Escherichia coli* ATCC 25922	196	
			*Staphylococcus aureus* ATCC 25923	49	
			BEC 9393 (MRSA)	49	
	*Cirsium setidens*	Spherical, 117.8 nm	*Bacillus cereus*	310	Shin et al. (2021)
			*Escherichia coli*	620	
			*Salmonella enterica*	620	
			*Staphylococcus aureus*	310	
	*Dillenia indica*	Oval, 50–900 nm	*Staphylococcus aureus* MTCC96	–	Krishnan et al. (2020)
	*Emblica officinalis*	Spherical, 15–40 nm	*Escherichia coli* MTCC 41	59.83	Gunti et al. (2019)
			*Listeria monocytogenes* MTCC 657	33.17	
			*Staphylococcus aureus* MTCC 96	9.16	
	*Ephedra aphylla*	Spherical and tetragonal, 13.95–26.26 nm	*Salmonella enterica* serotype Typhimurium ATCC 14028	–	El-Zayat et al. (2021)
			*Staphylococcus epidermidis* ATCC 12228	–	
			*Bacillus cereus* ATCC 11778	–	
			*Staphylococcus aureus* ATCC 6538	–	
			*Escherichia coli* ATCC 10536	–	
			*Listeria monocytogenes* ATCC 19115^™^	–	
	Ginger	Spherical, 100–150 nm	*Staphylococcus aureus*	150	Menon et al. (2019)
			*Escherichia coli*	150	
			*Proteus* sp.	150	
	Green orange	Spherical, 10–20 nm	MRSA	0.00494	Dang-Bao et al. (2022)
	*Moringa oleifera*	Spherical, 50–200 nm	*Listeria monocytogenes* ATCC 19112	700	Ao et al. (2022)
			*Corynebacterium diphtheriaec* CMCC 38017	70	
	Orange	Spherical, 16–95 nm	MDR *Escherichia coli*	50	Salem S. S. et al. (2022)
			*Staphylococcus aureus* ATCC 2921	25	
			MDR *Staphylococcus aureus*	25	
	Pepper	Spherical, 90.6 ± 14.4 nm	MDR *Escherichia coli*	72.2	Shah et al. (2022)
			MRSA	85.1	
	*Phyllanthus Emblica*	Spherical, ∼50.02 nm	*Escherichia coli*	16	Matai et al. (2020)
			*Staphylococcus aureus*	32	
	Prickly pear	Spherical, 10–87.4 nm	*Staphylococcus aureus* ATCC 25923	15.62	Hashem et al. (2022)
			*Escherichia coli* ATCC 25922	125	
	*Psidium guajava*	Spherical, 8–20 nm	*Escherichia coli* MTCC 405	23	Alam et al. (2018)
			*Staphylococcus aureus* MTCC 3160	11.7	
	*Saussurea costus*	Spherical, 2.21–11.63 nm	*Escherichia coli*	20.0	Al-Saggaf et al. (2020)
			*Salmonella enterica* serotype Typhimurium	17.5	
			*Staphylococcus aureus*	25.0	
	Tarragon	Quasi-spheres, 20–50 nm	*Bacillus cereus ATCC* 11778	1	Yilmaz et al. (2021)
			*Listeria monocytogenes* DSM2 15675	1	
			*Listeria monocytogenes* DSM2 19094	1	
			*Staphylococcus aureus* ATCC 29213	1	
	*Urtica dioic*	Spherical, 21.7–83.6 nm	*Escherichia coli* ATCC 25922	125	Hashem and Salem (2022)
			*Staphylococcus aureus* ATCC 25923	500	
	*Ziziphus spina-christi*	Spherica, 20–45 nm	*Escherichia coli* ATCC25922	–	lashin et al. (2021)
			*Staphylococcus aureus* ATCC25923	–	
Bacteria	*Bacillus licheniformis*	Spherical, 10–50 nm	*Bacillus cereus* DSMZ 345	20	Khiralla and El-Deeb (2015)
			*Staphylococcus aureus* ATCC 29213	20	
			*Escherichia coli* O157:H7 ATCC 43895	20	
			*Salmonella enterica* serotype Typhimurium ATCC 23564	20	
			*Salmonella enterica* serotype Enteritidis ATCC 4931	20	
	*Bacillus subtilis* AL43	Spherical, 32–86 nm	*Bacillus cereus*	100	Abdel-Moneim et al. (2022)
			*Staphylococcus aureus*	100	
			*Listeria monocytogenes*	100	
			*Escherichia coli*	100	
			*Salmonella enterica* serotype Typhimurium	100	
	*Providencia* sp. DCX	Spherical and pseudospherical, 46–333 nm	*Staphylococcus aureus*	10	Zhang et al. (2021)
			*Bacillus cereus*	10	
			*Escherichia coli*	10	
			*Vibrio parahemolyticus*	10	
	*Ralstonia eutropha*	Spherical, 40–120 nm	*Escherichia coli*	250	Srivastava and Mukhopadhyay (2015)
			*Staphylococcus aureus*	100	
Fungi	*Aspergillus quadrilineatus*	Spherical, 20–60 nm	*Bacillus cereus* ATCC10876	125	Hussein et al. (2022)
			*Staphylococcus aureus* ATCC6538	125	
			*Escherichia coli* ATCC11229	62.5	
	*Aspergillus ochraceus*	Spherical, 25–75 nm	*Bacillus cereus* ATCC10876	125	
			*Staphylococcus aureus* ATCC6538	125	
			*Escherichia coli* ATCC11229	62.5	
	*Aspergillus terreus*	Spherical, 10–80 nm	*Bacillus cereus* ATCC10876	250	
			*Staphylococcus aureus* ATCC6538	500	
			*Escherichia coli* ATCC11229	250	
	*Fusarium equiseti*	Spherical, 20–90 nm	*Bacillus cereus* ATCC10876	250	
			*Staphylococcus aureus* ATCC6538	500	
			*Escherichia coli* ATCC11229	250	
	*Penicillium corylophilum*	Spherical, 29.1–48.9 nm	*Escherichia coli* ATCC 8739	4.68	Salem et al. (2020)
			*Staphylococcus aureus* ATCC 6538	4.68	
	*Penicillium expansum* ATTC 36200	Spherical, 4–12.7 nm	*Staphylococcus aureus* ATCC23235	62.5	Hashem et al. (2021b)
			*Escherichia coli* ATCC8739	125	
	*Rhizopus oryzae* MG518370	Spherical, 20–200 nm	*Staphylococcus aureus* ATCC 6538	70	Abu-Elghait et al. (2021)
			*Escherichia coli* ATCC 8739	1000	
	*Streptomyces enissocaesilis*	Spherical, 20–211 nm	*Bacillus cereus*	49	Shaaban and El-Mahdy (2018)
			*Staphylococcus aureus* ATCC 29213	395	
			*Staphylococcus aureus* S1.1	49	
			MRSA 303	14.7	
			MRSA 402	60	
			MRSA 807	60	
			*Escherichia coli* ATCC 12435	197.5	
			*Escherichia coli* E7	197.5	
	*Streptomyces* sp. (M10A65)	Spherical, 20–150 nm	*Escherichia coli*	40	Ramya et al. (2019)
			*Staphylococcus aureus*	40	

### 2.1. Plant extract-based Bio-SeNPs

Research on green synthetic functional nanomaterials based on plants has attracted the attention of an increasing number of researchers (Naikoo et al., 2021). Plant extract-based Bio-SeNPs have the advantages of mild reaction, low cost, and easy operation (Bao et al., 2021). Furthermore, natural compounds are abundant in all parts of the plant, which provides suitable conditions for the synthesis of Bio-SeNPs (Jadoun et al., 2020; Ikram et al., 2021). In the synthesis of Bio-SeNPs, plant extracts provide three major groups of substances, including reducing agents, stabilizers, and capping agents (Abadi et al., 2022; Khan et al., 2022). In addition, plant extracts are abundant in antibacterial substances such as phenols, phenolic acids, terpenoids, and alkaloids (Alibi et al., 2021), which contribute to the synthesis and bioactivity of antibacterial nanomaterials.

Edible medicinal plants and agricultural waste are good choices among the plant sources for Bio-SeNP synthesis (Kumari et al., 2019; Jeevanandam et al., 2022). In particular, edible medicinal plants, combined with nanotechnology, have produced a number of extremely excellent nano antibacterial agents (Ghosh et al., 2021). *Costus* root extract was used as a reducing agent for the synthesis of antibacterial Bio-SeNPs against *Salmonella enterica* serotype Typhimurium, *E. coli*, and *S. aureus* with MICs of 17.5, 20.0, and 25.0 μg/mL, respectively (Al-Saggaf et al., 2020). Similarly, *Ephedra aphylla* aqueous extract was also used to synthesize Bio-SeNPs that inhibited *S. enterica* serotype Typhimurium, *E. coli*, *B. cereus*, *L. monocytogenes*, and *S. aureus*, containing phenolic, flavonoid and tannin compounds from *Ephedra aphylla* (El-Zayat et al., 2021). Bio-SeNPs produced by tarragon leaf extract could act against *B. cereus*, *E. coli*, *L. monocytogenes*, *S. aureus* and *Salmonella* spp. (Yilmaz et al., 2021). Additionally, Bio-SeNPs synthesized by ascorbic acid and *Cirsium setidens* extract could control *B. cereus*, *E. coli*, *Salmonella enterica*, and *S. aureus* (Shin et al., 2021).

Compared to edible-medicinal extract-mediated Bio-SeNPs, Bio-SeNPs based on agricultural waste took advantage of economy, which showed promising antibacterial results (Krishnani et al., 2022). Bio-SeNPs based on extracts of prickly pear peel waste (PPPW) demonstrated great antibacterial activity against *S. aureus* and *E. coli* (Hashem et al., 2022). Similarly, Bio-SeNPs from orange peel waste were resistant to *S. aureus*, MDR *S. aureus* and MDR *E. coli*, most sensitive to *S. aureus* with an MIC of 25 μg/mL and exhibited noticeable antibiofilm activity (Salem S. S et al., 2022). Additionally, Bio-SeNPs based on green orange peel could work against methicillin-resistant *S. aureus* with an MIC of 0.00494 μg/mL, mainly attributed to the higher polyphenol content of the orange peel extract (Dang-Bao et al., 2022). Both edible medicinal plants and agricultural waste-synthesized Bio-SeNPs have special features of their own, and edible medicinal plants probably have better antibacterial properties and safety, while agricultural waste is more affordable. Therefore, rational selection is essential to balance antibacterial activity and economic cost.

**Table 2:** *In vivo*/*vitro* assessment of Bio-SeNPs toxicity based on cells and animals.

Sources	Biological model (cell and animal)	Effects	Concentrations (μg/mL)	References
*Allium sativum* pulp	Normal Vero cells	Low toxicity	15–90	Anu et al. (2016)
*Cirsium setidens*	Normal mouse fibroblast cell line (NIH3T3)	Low toxicity	3.1–100	Shin et al. (2021)
	Human lung cancer cell line (A549)	High toxicity	3.1–100	
*Lactococcus lactis* NZ9000	Intestinal porcine enterocytes jejunum (IPEC-J2 cells)	No toxicity; Protect	64	Xu et al. (2019)
Lemon leaf	Lymphocytes	No toxicity; Protect	–	Prasad et al. (2013)
*Murraya koenigii* berry	RAW 264.7 macrophages	Low toxicity	10–90	Yazhiniprabha and Vaseeharan (2019)
*Ocimum tenuiflorum*	Human HEK293 cells	Low toxicity	50–200	Olawale et al. (2022)
*Portulaca oleracea*	Normal Vero cells	No toxicity	31.25–62.5	Fouda et al. (2022)
	Human normal lung fibroblast (WI-38)	No toxicity	31.25–62.5	
	Human hepatocellular carcinoma (HepG2)	Low toxicity	31.25–62.5	
*Penicillium expansum* ATTC 36200	Vero cell line CCL-81	Low toxicity	125–1000	Hashem et al. (2021b)
	Prostate cancer cell line (PC3)	High toxicity	31.25–1000	
*Penicillium corylophilum*	Human normal lung fibroblast (WI-38)	Low toxicity	31.25–1000	Salem et al. (2020)
	Human colorectal adenocarcinoma cells (cancer Caco-2)	High toxicity	31.25–1000	
*Spirulina platensis*	Normal kidney (Vero) cells	Low toxicity	0.39–100	Abbas et al. (2021)
	Transformed human liver epithelial-2 (THLE-2) cell lines	Low toxicity	0.39–100	
*Bacillus subtilis* MTCC441	Zebrafish embryos	Low toxicity	5–25	Zhang et al. (2021)
Lycopene	Rat	Low toxicity	0.5	Al-Brakati et al. (2021)
*Morinda citrifolia*	Brine Shrimp	Low toxicity	5–25	Nagalingam et al. (2022)
*Murraya koenigii* berry	*Artemia nauplii*	Low toxicity	10–50	Yazhiniprabha and Vaseeharan (2019)
Potato	Zebrafish embryos	Low toxicity	10–20	Chandramohan et al. (2019)
*Providencia* sp. DXC	Zebrafish	Low toxicity	0.5–3	Fan et al. (2022)

### 2.2. Bacteria-based Bio-SeNPs

Bacteria are considered biofactory for the synthesis of nanomaterials because they can efficiently transform toxic metals/nonmetals into useful nanomaterials (Mohanta et al., 2020; Spivak et al., 2020). Selenium-resistant bacteria can convert highly toxic selenite and selenate oxyanions into nontoxic Bio-SeNPs through a cellular detoxification mechanism (Ojeda et al., 2020). Meanwhile, bacterial synthesis of Bio-SeNPs is also a process of self-detoxification, and there are many proteins in the cell involved in this process (Tugarova and Kamnev, 2017). The mechanism of Bio-SeNPs synthesis by bacteria is complex, and further exploration is necessary for the specific synthesis mechanism (Escobar-Ramírez et al., 2021; Ullah et al., 2022). At present, many bacteria have been reported to synthesize Bio-SeNPs, such as *Bacillus licheniformis* (Khiralla and El-Deeb, 2015), *Bacillus subtilis* (Abdel-Moneim et al., 2022), *Streptomyces enissocaesilis* (Shaaban and El-Mahdy, 2018), *Providencia* sp. (Zhang et al., 2021), *Streptomyces* sp. (Ramya et al., 2019), and *Ralstonia eutropha* (Srivastava and Mukhopadhyay, 2015), which could inhibit various foodborne pathogens. However, some bacteria might carry toxins or other harmful factors (Abebe et al., 2020), so it is necessary to select harmless selenium-resistant bacteria to synthesize safer Bio-SeNPs.

Fortunately, probiotics possess multiple benefits to human health and are considered factories for the production of Bio-SeNPs (Kerry et al., 2018; Yang and Yang, 2023), which is advantageous to the food industry. Bio-SeNPs synthesized by *Lactobacillus pentosus* ADET MW861694 were used to control foodborne pathogens such as *Salmonella enterica* subsp. *arizonae*, *E. coli*, *S. enterica* serotype Typhimurium, and *S. aureus* (Christianah Adebayo-Tayo et al., 2021). Similarly, Bio-SeNPs synthesized by *Lactobacillus sporogenes* were used to inhibit *S. aureus* and *E. coli* (Kaur et al., 2018). Furthermore, in *Lactobacillus acidophilus,* extracellularly synthesized Bio-SeNPs were reported against the drug-resistant bacteria *S. aureus* and *E. coli* to inhibit biofilms (Alam et al., 2019). Currently, research on probiotic bacteria-synthesized Bio-SeNPs against foodborne microorganisms is relatively scarce and requires further study.

### 2.3. Fungi-based Bio-SeNPs

Fungi possess high metal tolerance and abundant metabolites, which are powerful tools for the synthesis of biogenic nanomaterials (Adebayo et al., 2021; Sonawane et al., 2022). Recently, some fungi have been used to synthesize Bio-SeNPs, such as *Mariannaea* sp. HJ (Zhang et al., 2019), *Aureobasidium pullulans*, *Mortierella humilis*, *Trichoderma harzianum* and *Phoma glomerata* (Liang et al., 2019), and *Aspergillus quadrilineatus*, *Aspergillus ochraceus*, *Aspergillus terreus*, and *Fusarium* *equiseti* (Hussein et al., 2022). Furthermore, fungal synthesis of Bio-SeNPs has great antibacterial potential. Bio-SeNPs synthesized by *Monascus purpureus* could perform against *S. aureus* and *E. coli* with an MIC of 100 μg/mL (El-Sayed et al., 2020) and against *A. acidoterrestris* with an MIC of 3,000 μg/mL (Sun et al., 2021). In particular, the Bio-SeNPs synthesized by some *Penicillium* spp. demonstrated formidable antibacterial abilities. Bio-SeNPs synthesis from *Penicillium chrysogenum* PTCC 5031 could inhibit *S. aureus* and *L. monocytogenes* (Vahidi et al., 2020). Bio-SeNPs produced by *Penicillium corylophilum* could operate against *E. coli* and *S. aureus* with MICs of 9.37 μg/mL and 37.5 μg/mL, respectively (Salem et al., 2020). Bio-SeNPs synthesized by *Penicillium expansum* ATTC 36200 could control *S. aureus* and *E. coli* (Hashem et al., 2021b). However, *Penicillium* spp. might produce antibiotics such as penicillin (Kumar et al., 2018), resulting in limited applications within the food industry.

Bio-SeNPs produced by fermentation of edible mycelium and yeast were safer and may be more promising in the food industry. Edible *Lentinula edodes* could be used to synthesize Bio-SeNPs, and mycelium reddening (Tsivileva et al., 2012) and accumulation of Bio-SeNPs (Vetchinkina et al., 2013) were observed during mycelial growth. Additionally, *Saccharomyces cerevisiae* extract was used to synthesize Bio-SeNPs, and it showed excellent antibacterial activity against *S. aureus* and *E. coli* (Salem, 2022). However, there are fewer studies on the synthesis of antibacterial Bio-SeNPs from edible mycelium and yeast, which may have better applications in the food industry.

## 3. Antibacterial mechanisms of Bio-SeNPs

**Figure 2:**
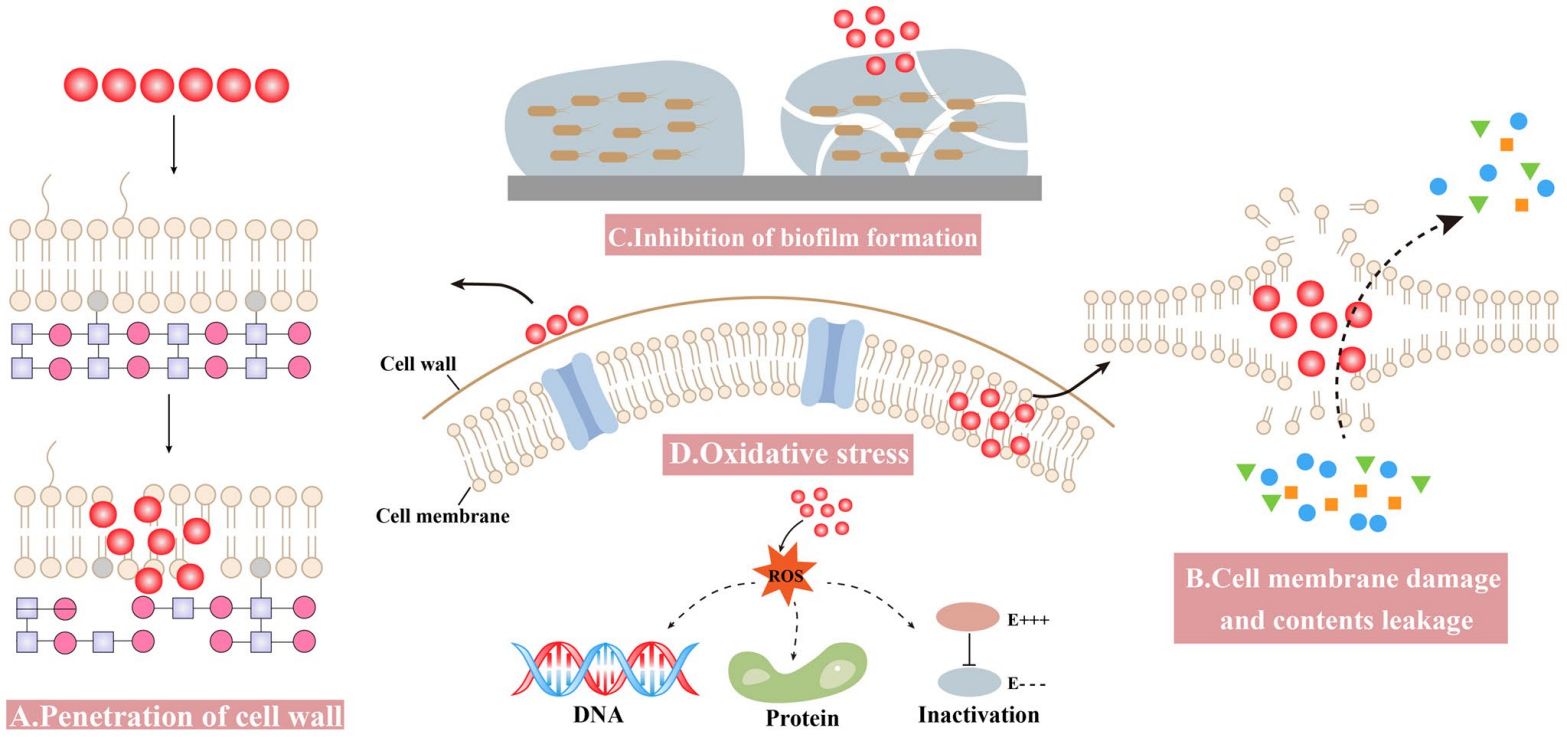
Antibacterial mechanisms of Bio-SeNPs. **(A)** Penetration of the cell wall (Alam et al., 2019; Cittrarasu et al., 2021; Zhang et al., 2021). **(B)** Cell membrane damage and contents leakage (Tareq et al., 2018; Sun et al., 2021; Prasathkumar et al., 2022). **(C)** Inhibiting the formation of biofilm (Ramya et al., 2015; Shakibaie et al., 2015; Miglani and Tani-Ishii, 2021; Haddadian et al., 2022; Ullah et al., 2023). **(D)** Oxidative stress (Cremonini et al., 2018; Alam et al., 2019; Prasathkumar et al., 2022).

The antibacterial mechanism of nanomaterials is complex due to various attributes (Matai et al., 2020). Some general mechanisms are summarized as follows: (1) penetration of the cell wall, (2) cell membrane damage and contents leakage, (3) inhibiting the formation of biofilm, and (4) inducing oxidative stress (Figure 2).

### 3.1. Penetration of the cell wall

Bio-SeNPs bind to the cell wall and further affect the integrity of cell membranes and cell morphology. Nanoparticles can anchor to Singh et al. (2014) and/or burrow into bacterial cell walls (Galbadage et al., 2019), causing structural changes in cell membrane permeability and leading to bacterial death. Compared to gram-negative bacteria, the thicker peptidoglycan structure of the gram-positive bacterial cell wall might make it more resistant to drugs (Reygaert, 2018; Impey et al., 2020; Pasquina-Lemonche et al., 2020). Bio-SeNPs synthesized by *Providencia* sp. DCX exhibited concentration-dependent inhibition against five pathogenic bacteria, including G^+^ (*S. aureus* and *B. cereus*) and G^−^ (*Pseudomonas aeruginosa*, *Vibrio parahemolyticus* and *E. coli*). Bio-SeNPs were more lethal to gram-negative bacteria, probably due to the thin peptidoglycan of G^−^ bacteria, and selenium nano could more easily penetrate their cell walls and disrupt the integrity of cell membranes (Zhang et al., 2021). Bio-SeNPs produced by *L. acidophilus* inhibited pathogens such as *Klebsiella pneumoniae* and *P. aeruginosa*, with much lower MIC values compared to gentamicin. The lower MIC values of Bio-SeNPs might be due to the electrostatic interactions responsible for Bio-SeNPs adhesion to the bacterial cell wall, causing bacterial death (Alam et al., 2019). Meanwhile, *Ceropegia bulbosa* Roxb extract-based Bio-SeNPs could inhibit bacteria such as *B. subtilis* and *E. coli*. It is possible that ionic interactions caused the negatively charged Bio-SeNPs to bind to the bacterial surface, blocking the synthesis of bacterial cell walls (Cittrarasu et al., 2021).

### 3.2. Cell membrane damage and contents leakage

Bio-SeNPs may disrupt cell membrane integrity and cause leakage of cytoplasmic contents. A biophysical model for the interaction of nanomaterials with bacterial cell membranes has been proposed, which suggests that adsorption of NPs leads to membrane stretching and squeezing, causing cell rupture and death (Linklater et al., 2020). The use of Bio-SeNPs to disrupt bacterial cell wall integrity and cause leakage of contents was considered an effective strategy (Makabenta et al., 2021). Bio-SeNPs synthesized in *M. purpureus* were used to assess the inhibition of *A. acidoterrestris* (Sun et al., 2021). The SEM results showed that 3,000 μg/mL Bio-SeNPs caused the bacterial cells to shrink slightly, and the surface became rough with holes and wrinkles, while the bacterial cells were damaged with severe distortion and irregularity when the concentration was increased to 5,000 μg/mL. Meanwhile, further determination of the leakage of cellular contents was performed. Bacteria treated with Bio-SeNPs showed a significant amount of leakage of protein, DNA and RNA. *Azadirachta indica* leaf aqueous extract was used to synthesize Bio-SeNPs against *Clostridium botulinum* (Tareq et al., 2018). The SEM results showed that the bacteria treated with 100 μg/mL Bio-SeNPs were severely damaged, misshapen and fragmentary. Moreover, after 4 h of Bio-SeNPs treatment, the bacteria leaked more reducing sugars and proteins. It was revealed that Bio-SeNPs can disrupt cell membranes and accelerate the leakage of reducing sugars and proteins from bacteria. The *Senna auriculata* flower and leaf aqueous extract was used to synthesize Bio-SeNPs against *B. subtilis*, *MRSA*, *E. coli*, and *P. aeruginosa* (Souza et al., 2022). Optical microscopy results showed that Bio-SeNPs inhibited pathogenic bacteria, and FESEM results also showed the deposition of Bio-SeNPs on the cell surface, causing bacterial rupture. Furthermore, protein and reducing sugar leakage was detected after treatment with 250 μg/mL Bio-SeNPs.

### 3.3. Inhibition of biofilm formation

The inhibition of biofilm formation and subsequent growth inhibition is another antibacterial mechanism of Bio-SeNPs. Bio-SeNPs synthesized by *Lysinibacillus* sp. NOSK effectively inhibited *P. aeruginosa* biofilm formation, and its large surface area, small size and spherical shape may be an important factor (San Keskin et al., 2020). Bio-SeNPs produced by *Bacillus subtilis* BSN313 were strongly bound to bacterial surfaces and destroyed bacterial cells by disintegrating the membranes of *P. aeruginosa*, *S. enterica* serotype Typhimurium and *S. aureus* (Ullah et al., 2023). In addition, Bio-SeNPs (2 μg/mL) generated by *Bacillus* sp. MSh-1 had strong adhesion to biofilm-producing bacteria and inhibited the biofilm formation of *S. aureus*, *P. aeruginosa*, and *Proteus mirabilis* (Shakibaie et al., 2015). Bio-SeNPs (1,000 μg/mL) synthesized by fresh guava leaves inhibited the growth of biofilm formation, and the carbohydrate and protein concentrations of the treated *Enterococcus faecalis* biofilm decreased by approximately 73 and 71%, respectively (Miglani and Tani-Ishii, 2021). Bio-SeNPs also have a better inhibitory effect on many biofilms forming multidrug resistant bacteria. For instance, Bio-SeNPs produced by *Streptomyces minutiscleroticus* M10A62 could effectively inhibit biofilm formation of six biofilm-forming multidrug-resistant strains of *Acinetobacter* (4,117, 1,677, 2,030, 674, 2,020, and 1,370) (Ramya et al., 2015). In addition, the *Trifolium cherleri* aerial aqueous extract was used to synthesize Bio-SeNPs for anti-biofilm of *S. aureus*, *E. faecalis*, *E. coli*, and *P. aeruginosa* (Souza et al., 2022). Further analysis of the expression levels of biofilm-related genes such as *icaD*, *Ace*, *fmH*, and *pelf* revealed that the expression levels of related genes were significantly reduced in bacteria treated with Bio-SeNPs (Souza et al., 2022). This result suggested that Bio-SeNPs might bind to transcription factors and repress the expression of biofilm-related genes.

### 3.4. Oxidative stress

Bio-SeNPs induce high ROS production, break ROS homeostasis and cause oxidative stress. Many nanomaterials produce excess ROS, leading to various injuries, such as membrane disabilities, mitochondrial damage, and destruction of nucleic acids and proteins (Sadoq et al., 2023). In addition, the large amount of ROS disrupted the antioxidant system of bacteria and severely limited their viability (Mourenza et al., 2020). Many studies have shown that ROS produced by selenium nano exhibited effective antibacterial ability (Sakr et al., 2018; Kondaparthi et al., 2019; Bisht et al., 2022). The ROS assay results indicated that *Senna auriculata* extract-produced Bio-SeNPs entered the bacterial cell, causing a rapid increase in fluorescence intensity from intracellular ROS production, resulting in oxidative stress damage and contributing to bacterial death (Souza et al., 2022). Similarly, Bio-SeNPs synthesized by *Stenotrophomonas maltophilia* SeITE02 might kill bacteria by producing ROS (Cremonini et al., 2018). The intracellular ROS production of *P. aeruginosa* PAO1, *S. aureus* Mu50 and *Burkholderia cenocepacia* LMG16656 increased after treatment with Bio-SeNPs, while the survival of these strains was significantly limited (Cremonini et al., 2018). In *L. acidophilus*, the synthesized Bio-SeNPs could also control bacteria by producing ROS (Alam et al., 2019). The expression levels of superoxide dismutase (SOD) and catalase were substantially induced by ROS in *E. coli*, *S. aureus*, *B. subtilis*, *P. aeruginosa*, *and K. pneumoniae* after treatment with Bio-SeNPs (Alam et al., 2019).

## 4. Bio-SeNPs antibacterial applications in the food industry

**Figure 3:**
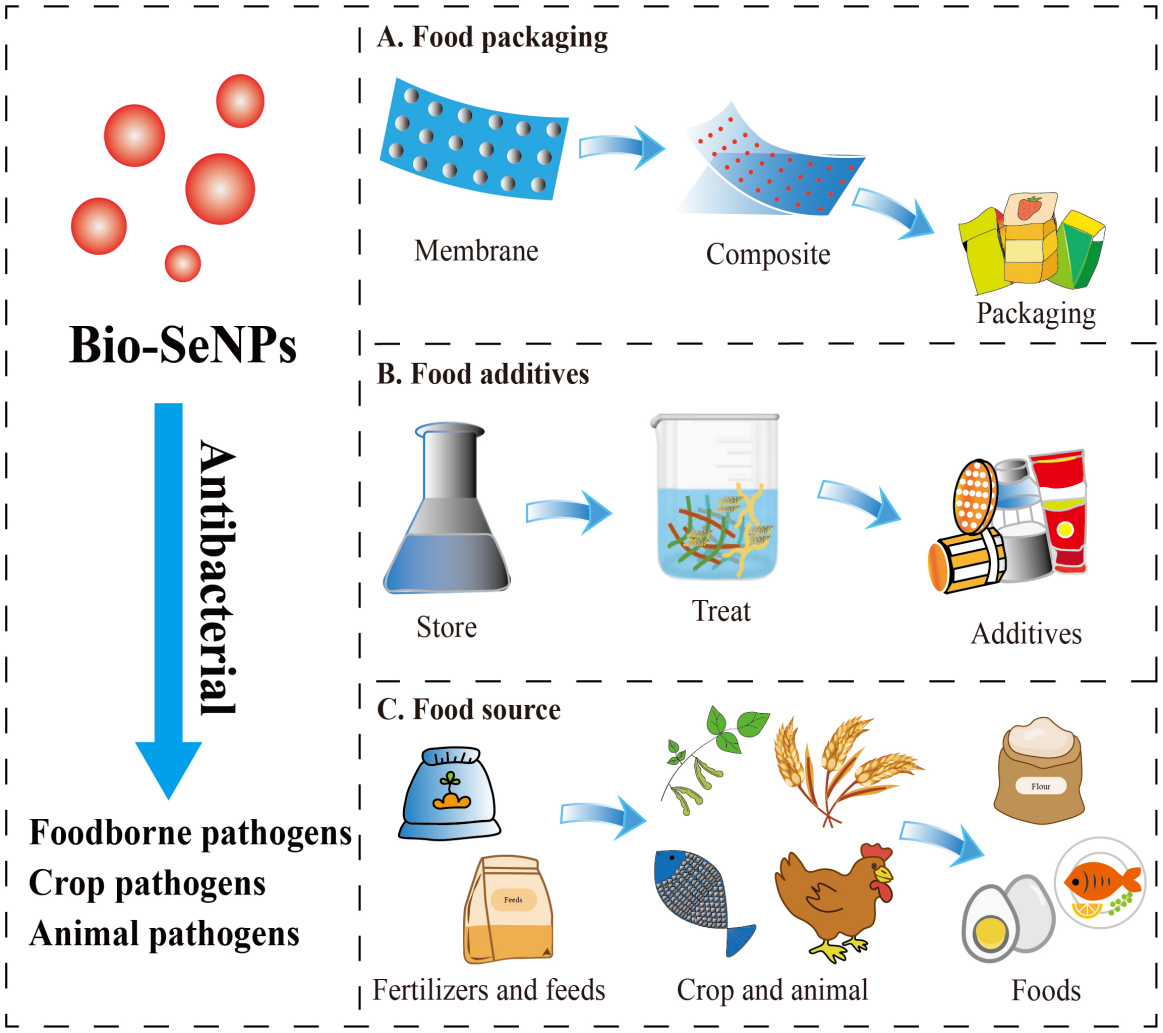
The potential application forms of antibacterial Bio-SeNPs in the food industry. **(A)** Bio-SeNPs were combined with membrane materials to form composite food packaging film (Jamróz et al., 2019a,b; Lu et al., 2020; Alghuthaymi et al., 2021; Ndwandwe et al., 2022). **(B)** Bio-SeNPs were used as food additives (Sun et al., 2021; Ao et al., 2022; Saad et al., 2022). **(C)** Bio-SeNPs were used as fertilizers/feeds for crops and animals (Hu et al., 2019; Ayoub et al., 2021; El-Saadony et al., 2021; Hashem et al., 2021a; Sarkar and Kalita, 2022; Shahbaz et al., 2022; Taha et al., 2023).

Food packaging and food additives are used to solve the contamination of food-borne pathogens. Nanomaterial-based food packaging and additives exhibit great potential in food antibacterial applications. Although various nanomaterial-based food packaging and additives have shown excellent antibacterial ability, their application might be limited by toxicity or nonedible components, which could result in food safety problems (Chaudhry et al., 2010). Fortunately, selenium is an essential trace element for the human body (Yang et al., 2022), with promising application prospects in the Bio-SeNPs form as depicted (Figure 3).

Several studies have proven that Bio-SeNPs can be used as food packaging material to extend shelf life. Jamróz and group developed furcellaran-gelatin films with SeNPs and AgNPs, that possessed great antibacterial activity against *S. aureus*, MRSA and *E. coli* (Jamróz et al., 2019a). The packaging system could extend the shelf life of mini kiwi (Jamróz et al., 2019a). Similarly, the SeNPs and natural extract-modified furcellaran film showed excellent antibacterial activity against *S. aureus*, MRSA and *E. coli* and showed great potential applications in fish products shelf life (Jamróz et al., 2019b). Selenium microparticles and polylactic acid–based films also showed noticeable inhibition of *S. aureus* and *E. coli* (Lu et al., 2020). Alghuthaymi et al. (2021) developed coatings based on chitosan and cinnamon extract synthesized Bio-SeNPs that had antibacterial activities against *E. coli*, *S. enterica* serotype Typhimurium, *S. aureus*, and *L. monocytogenes*, which are potential edible coating (EC) basements (Alghuthaymi et al., 2021). Bio-SeNPs were also found to enhance the activity of potato starch films. SeNPs/potato starch nanofilm exhibited an inhibitory effect on *S. enterica* serotype Typhimurium, *E. coli* and *B. cereus* (Souza et al., 2022). All these studies indicated that Bio-SeNPs could be used as active food packaging material in replacement of the traditional material.

At present, Bio-SeNPs are rarely reported to be added directly to foods as antibacterial agents. Bio-SeNPs produced by *M. purpureus* showed the ability to inhibit *A. acidoterrestris*, which is an acid-resistant and heat-resistant bacterium that causes fruit juice spoilage (Sun et al., 2021). In our previous study, Bio-SeNPs generated by *Moringa oleifera* could efficiently clear *L. monocytogenes* on raw salmon (Ao et al., 2022). More interestingly, Bio-SeNPs synthesized by *Bacillus subtilis* AS12 could decrease the accumulation of heavy metals and pathogenic microbes in fish organs while improving growth performance (Souza et al., 2022). These reports suggested that Bio-SeNPs have the potential to be used as food additives or additives for food-derived animal culture for better antibacterial activity and extended shelf life.

In addition, Bio-SeNPs were used to control the crop and animal pathogens and supply the selenium element in foods. Bio-SeNPs (100 μg/mL) synthesized by *Bacillus cereus* showed an 85.1% reduction on mycelial growth of *Alternaria alternata*, which could effectively control leaf spot disease caused by *Alternaia alternata* in common beans and also improve plant growth and yield (Taha et al., 2023). Similarly, Bio-SeNPs produced by *Bacillus megaterium* ATCC 55000 could effectively inhibit the growth of *Rhizoctonia solani* RCMB 031001 to reduce root rot, improve morphological and metabolic indicators, and increase yield (Hashem et al., 2021a). *Trichoderma harzianum*-derived Bio-SeNPs (200 μg/mL) could significantly inhibit *Alternaria alternata* XJa1, *Fusarium verticillioide* BJ6 and *Fusarium graminearum* PH1 to protect corn and pears (Hu et al., 2019). Additionally, Bio-SeNPs were also used to suppress *Triticum aestivum* L. crown and root rot diseases induced by *Fusarium* species (El-Saadony et al., 2021), control stripe rust disease on *Triticum aestivum* L. (Souza et al., 2022), and promote the growth of mustard (Sarkar and Kalita, 2022). Likewise, Bio-SeNPs were exhibited excellent antibacterial against animal pathogens (Gad et al., 2022). Bio-SeNPs synthesized by *Citrullus colocynthis* extract could reduce mortality after *Aeromonas sobria* infection and improved immune function, antioxidant capacity and disease resistance in *Oreochromis niloticus* (Ayoub et al., 2021). Bio-SeNPs produced by *Lactobacillus delbrueckii* subsp. *bulgaricus* (NCAIM B 02206) were also used as feed additives for effective supplementation in *O. niloticus* diets to improve growth, oxidative status and immune-related gene expression (Dawood et al., 2020). Additionally, Bio-SeNPs were reported to promote the growth of *Macrobrachium rosenbergii* (Satgurunathan et al., 2023), and improve broiler performance and intestinal integrity (Ali et al., 2022). Overall, Bio-SeNPs may be a promising material to antibacterial against crop and animal pathogens, as well as contribute to the growth of crops and animals, and supply selenium element.

## 5. Toxicity of Bio-SeNPs

**Figure 4:**
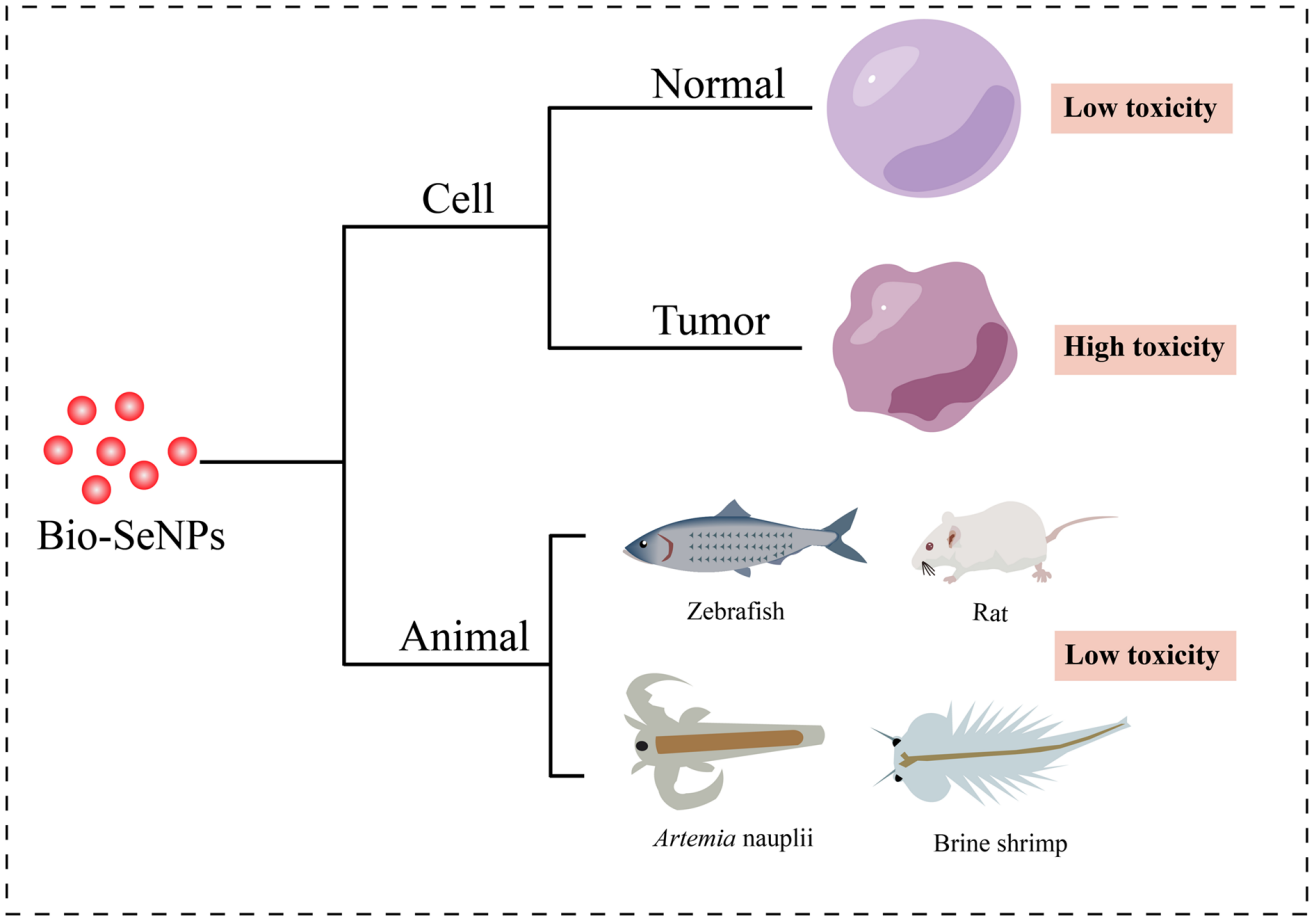
Toxicity of Bio-SeNPs in cells and animals. Cytotoxicity of Bio-SeNPs on normal cells (Prasad et al., 2013; Anu et al., 2016; Xu et al., 2019; Yazhiniprabha and Vaseeharan, 2019; Abbas et al., 2021; Fouda et al., 2022; Olawale et al., 2022) and tumor cells (Ranjitha and Ravishankar, 2018; Rajkumar et al., 2020; Salem et al., 2020; Shin et al., 2021; Hashem et al., 2021b; Al-Otaibi et al., 2022; Fouda et al., 2022). Toxicity of Bio-SeNPs on zebrafish (Chandramohan et al., 2018, 2019; Fan et al., 2022), rats (Al-Brakati et al., 2021), *Artemia nauplii* (Yazhiniprabha and Vaseeharan, 2019), and brine shrimp (Nagalingam et al., 2022).

As a result of their great biological activities and potential applications, the toxicity of Bio-SeNPs has attracted increasing attention (Figure 4).

### 5.1. Cytotoxicity of Bio-SeNPs

Cytotoxicity assessment provides an essential foundation for the usage of Bio-SeNPs in the food industry. Various cells were used to test the toxicity of different biogenic selenium nanoparticles (Table 2). Bio-SeNPs produced by *Spirulina platensis* exhibited minimal cytotoxicity to normal kidney (Vero) cells and transformed human liver epithelial-2 (THLE-2) cell lines at concentrations of 0.39–100 μg/mL (Abbas et al., 2021). Similarly, 31.25–62.5 μg/mL Bio-SeNPs synthesized by *Portulaca oleracea* were almost nontoxic to Vero normal cells and human normal lung fibroblast (WI-38) lines (Fouda et al., 2022). Interestingly, Bio-SeNPs synthesized by *Lactococcus lactis* NZ9000 were not only nontoxic to intestinal porcine enterocytes jejunum (IPEC-J2) cells but could also alleviate enterotoxigenic *E. coli* K88-induced cell injury (Xu et al., 2019). In addition, Bio-SeNPs synthesized by lemon leaf extract protected lymphocytes, prevented DNA damage and reduced reactive oxygen species toxicity under UVB irradiation (Prasad et al., 2013). On the other hand, Bio-SeNPs also showed low cytotoxicity in some studies. Bio-SeNPs produced by *Ocimum tenuiflorum* revealed low toxicity to human embryonic kidney (HEK293) cells (Olawale et al., 2022). Bio-SeNPs (10–50 μg/mL) synthesized from *M. koenigii* berries exhibited low cytotoxicity on mouse mononuclear macrophages cells (RAW 264.7 macrophages), and minor cell destruction was observed at 50 μg/mL (Yazhiniprabha and Vaseeharan, 2019). The CC50 of Bio-SeNPs synthesized from *Allium sativum* pulp extract was 31.8 ± 0.6 μg/mL for Vero cells, while the CC50 of chemically synthesized SeNPs was 18.8 ± 0.8 μg/mL (Anu et al., 2016). These results suggested that the toxicity of Bio-SeNPs was lower than that of chemically synthesized SeNPs. Bio-SeNPs from different sources exhibited varied thresholds of toxicities depending on the dosage and constituents of the Bio-SeNPs.

Interestingly, Bio-SeNPs seem to exhibit higher toxicity to cancer cells than to normal cells. Bio-SeNPs synthesized by *Cirsium setidens* extracts were nontoxic to a normal mouse fibroblast cell line (NIH3T3) in the low concentration range (3.1–100 μg/mL) but significantly toxic to human non-small cell lung cancer (A549) cells (Shin et al., 2021). Likewise, Bio-SeNPs synthesized using *P*. *corylophilum* were less toxic to human normal lung fibroblasts (WI-38) than to human cancer colorectal adenocarcinoma epithelial cells (Caco-2) (Salem et al., 2020). Bio-SeNPs (31.25–1,000 μg/mL) from *P. expansum* ATTC 36200 also showed low toxicity to the Vero cell line CCL-81 but high toxicity to the human prostate cancer (PC3) cell line (Hashem et al., 2021b). Similar anticancer activity was also observed in human hepatocellular carcinomas HepG2 cells (Souza et al., 2022), human mammary tumor MCF-7 cells (Souza et al., 2022), human cervical carcinoma HeLa cells (Rajkumar et al., 2020) and human colorectal adenocarcinoma HT-29 cells (Ranjitha and Ravishankar, 2018). Compared to normal cells, Bio-SeNPs may be more inclined to counteract the rapid tumor cell proliferation and release more ROS to suppress tumor cells (Cui et al., 2018; Menon and Shanmugam, 2019). Accordingly, Bio-SeNPs might be great tumor agents.

### 5.2. Animal toxicity of Bio-SeNPs

It is critical to carry out animal toxicity tests before using Bio-SeNPs in the food industry. Researchers have used zebrafish, *Artemia nauplii*, shrimp and rats to test the toxicity of Bio-SeNPs. Zebrafish embryos treated with Bio-SeNPs synthesized by potato extract showed less toxicity at concentrations of 10–20 μg/mL, but exhibited improper heartbeat and edema of the embryonic sac, eye and head at concentrations of 30–50 μg/mL (Chandramohan et al., 2019). Similarly, Bio-SeNPs produced by *B. subtilis* MTCC441 were nontoxic to zebrafish embryos at 5 μg/mL, with low mortality at 10 μg/mL, but caused low heart rate, delayed hatching and low survival at 15–25 μg/mL (Chandramohan et al., 2018). Bio-SeNPs synthesized from *Providencia* sp. DXC had lower toxicity with an LC_50_ of 1.668 μg/mL at 96 h, whereas the chem-SeNPs caused more significant injury to liver and gill cells of zebrafish (Souza et al., 2022). Meanwhile, the LC50 and LC90 values of 68.27 μg/mL and 121.75 μg/mL for *A. nauplii* treated with Bio-SeNPs based on *M. koenigii* berry extracts, also showed slight toxicity while Bio-SeNPs accumulation was observed in the region of the median eye and food groove/gut, but damage to appendages and carapace was not evident (Yazhiniprabha and Vaseeharan, 2019). In addition, the survival ratio of brine shrimp treated with *Morinda citrifolia-*mediated Bio-SeNPs (5, 10, and 25 μg/mL) was 70, 80 and 30% within 2 days, respectively (Nagalingam et al., 2022). Interestingly, biosynthesized Lycopene-coated Bio-SeNPs (0.5 mg/kg) showed no significant toxicity to the liver and kidney organs and hematological parameters of rats, and even exhibited nephroprotective activity against AKI (glycerol-treated)-caused tissue damage in rat models (Al-Brakati et al., 2021). Different sources of biological selenium nanoparticles showed different toxic effects on different animals. Overall, the toxicity of Bio-SeNPs to animals is low, but it is essential to perform toxicity evaluation before any Bio-SeNPs are applied in food.

## 6. Outlook

In this review, we summarize the great potential of Bio-SeNPs for the control of foodborne pathogens and analyze the antibacterial application and safety in the food industry. Currently, microbes and plant extracts are being explored on large scale for the synthesis of Bio-SeNPs. Microbes and plants contribute various bioactive substances which are thought to confer higher antibacterial potential to these Bio-SeNPs. The Bio-SeNPs were applied in food additives, food packaging and fertilizers/feeds for crop and animal. In addition, some cellular and animal toxicity assessment experiments have shown that Bio-SeNPs are non-toxic/low toxicity at low antibacterial concentrations. It implied that Bio-SeNPs showed great potential in the application of food industry.

Even though Bio-SeNPs exhibit excellent application prospect, there is still lots of work to do before its application. (1) Due to the diversity of Bio-SeNPs synthesis processes and the complexity components, there are some uncontrollable factors in the actual production. So, it is more essential to choose a safe biological system to synthesize Bio-SeNPs with excellent antibacterial properties and higher economic value. Probiotics and edible fungi may be good choices. (2) The antibacterial mechanisms of Bio-SeNPs are not very deep yet, mainly focusing on the description of antibacterial phenomena. Further researches should be paid to the genetic level and focus on the relationship between the properties of Bio-SeNPs and their mechanisms and pathways of antibacterial activity. (3) At present, Bio-SeNPs are mainly used in food packaging materials or fertilizers for crop in some cases. However, the application forms of Bio-SeNPs need to be further developed for maximum benefits. (4) The toxicity analysis showed that Bio-SeNPs were either low toxic or nontoxic at low concentrations while their antibacterial activities were evident at high concentrations. Accordingly, the activities of Bio-SeNPs need to be further strengthened.

## Author contributions

BA, QD, and DL prepared the draft manuscript and the figures. XX, JT, and XS revised the manuscript. All authors contributed to the article and approved the submitted version.

## Funding

This study was supported by the Hubei Province Key R&D Program Project (2022BCE010), the National Natural Science Foundation of China (32000066), Hubei Province Central Government Guides Local Project, Natural Science Foundation of Hubei Province (2022CFB503), the Innovation Team Project of Hubei Education Department (T2022010), and the Open Foundation of the Hubei Key Laboratory of Edible Wild Plants Conservation and Utilization (EWPL202209).

## Conflict of interest

The authors declare that the research was conducted in the absence of any commercial or financial relationships that could be construed as a potential conflict of interest.

## Publisher’s note

All claims expressed in this article are solely those of the authors and do not necessarily represent those of their affiliated organizations, or those of the publisher, the editors and the reviewers. Any product that may be evaluated in this article, or claim that may be made by its manufacturer, is not guaranteed or endorsed by the publisher.
